# Blood Urea Nitrogen/Creatinine Ratio in Cushing’s Syndrome: Associations With Disease Status and Postoperative Changes

**DOI:** 10.1155/ije/2517065

**Published:** 2026-06-12

**Authors:** C. Yarkutay Turkkan, M. M. Canat, H. Erhan Gol, Y. Altuntas

**Affiliations:** ^1^ Department of Internal Medicine, Şişli Hamidiye Etfal Training and Research Hospital, Istanbul, Turkey, sislietfal.gov.tr; ^2^ Department of Internal Medicine, Hamidiye Faculty of Medicine, University of Health Sciences, Istanbul, Turkey, akdeniz.edu.tr; ^3^ Department of Endocrinology and Metabolism, Şişli Hamidiye Etfal Training and Research Hospital, Istanbul, Turkey, sislietfal.gov.tr; ^4^ Department of Endocrinology and Metabolism, University of Health Sciences, Istanbul, Turkey, akdeniz.edu.tr

**Keywords:** adenoma, BUN, Cushing’s syndrome, diagnosis, hypercortisolism

## Abstract

**Objective:**

Although various tests are used for Cushing’s syndrome (CS) screening and diagnosis, none exhibit ideal sensitivity or specificity. This study evaluates the association of the blood urea nitrogen (BUN)/creatinine ratio with disease status in CS and subclinical Cushing’s syndrome (SCS), and with postoperative changes in patients with CS.

**Design:**

A single‐center, observational, cross‐sectional study conducted at Endocrinology and Metabolic Diseases Clinic of a tertiary training and research hospital in Istanbul, Turkey (2017–2022).

**Patients:**

This study included 229 individuals: 151 CS/SCS patients and 78 healthy controls. CS cases were classified as adrenocorticotropic hormone (ACTH)–dependent (*n* = 52) or ACTH‐independent (*n* = 34).

**Measurements:**

BUN/creatinine ratios were compared among CS, SCS, and control groups, as well as between pre‐ and posttreatment states in CS patients. Correlations between BUN/creatinine ratio and standard CS diagnostic tests were analyzed. Receiver operating characteristic (ROC) analysis was performed to evaluate the discriminatory performance of the BUN/creatinine ratio between CS and control groups.

**Results:**

BUN/creatinine ratios were significantly higher in CS, ACTH‐independent CS, and SCS groups compared to controls. Cutoff values were > 15.34 for CS, > 13.67 for ACTH‐independent CS, and > 19.8 for SCS. The BUN/creatinine ratio correlated positively with basal plasma cortisol, 1 mg dexamethasone suppression test (DST), and midnight serum cortisol in ACTH‐dependent CS (*p* = 0.047, 0.038, 0.004). In all CS patients, it correlated with 1‐mg DST and midnight serum cortisol (*p* = 0.041, 0.049). In ACTH‐dependent CS, a positive correlation was found between pituitary adenoma diameter and the preoperative BUN/creatinine ratio (*p* = 0.036).

**Conclusions:**

The BUN/creatinine ratio may provide supportive information associated with CS status and postoperative changes in patients with CS, particularly when standard tests are unavailable or inconclusive. Larger multicenter studies are needed to confirm its clinical utility.

## 1. Introduction

Cushing’s syndrome (CS) is a chronic and potentially life‐threatening disorder characterized by excess corticosteroid exposure. While exogenous corticosteroid use remains the most frequent cause, endogenous CS, resulting from cortisol hypersecretion, is rare and arises from various etiologies. Chronic hypercortisolemia is associated with significant morbidity and increased mortality if left untreated [1]. Due to the lack of pathognomonic symptoms, biochemical confirmation is essential when clinical suspicion arises. First‐line tests include 1‐mg dexamethasone suppression test (DST), 24‐h urinary free cortisol (UFC), midnight salivary cortisol, and midnight serum cortisol levels. Once confirmed, further tests are required to determine CS etiology [2].

Considering the diversity of its symptoms and manifestations, the comorbidities it causes, and the associated increase in mortality, it is a complex disease that must be carefully considered in differential diagnosis when necessary [1]. Although numerous tests have been employed in screening and diagnostic processes to date, none have proven to be the ideal test due to the inability to achieve uniform sensitivity and specificity across all patients, high costs, the need for medication, additional clinical applications, or even hospitalization. The challenges in the diagnostic process are particularly evident in primary and secondary healthcare centers [3]. None of the first‐line tests possess optimal sensitivity or specificity. The search for an easily measurable, ideal diagnostic test continues [4].

Cortisol directly affects carbohydrate, lipid, and protein metabolism [5]. It increases catabolism in muscle, skin, and connective tissue while promoting insulin resistance in muscle tissue, leading to muscle atrophy through type 2 muscle fibers [6, 7]. In cases of glucocorticoid excess, this process can be explained by the inhibition of amino acid reuptake, resulting in reduced skeletal muscle growth and increased proteolysis [7]. As a consequence of muscle catabolism, urea production increases, leading to an elevated urea/creatinine ratio [8]. Therefore, in hypercortisolemic states, increased protein catabolism leads to increased urea production, while creatinine production decreases, resulting in an elevated blood urea nitrogen (BUN)/creatinine ratio. Plasma BUN and creatinine levels are easily accessible, cost‐effective tests that can be routinely performed even in primary healthcare centers.

This study aims to evaluate the association between the plasma BUN/creatinine ratio and disease status in patients with CS and subclinical Cushing’s syndrome (SCS), and to examine postoperative changes in patients with CS. Secondly, it aims to explore whether the BUN/creatinine ratio differs between adrenocorticotropic hormone (ACTH)‐dependent and ACTH‐independent CS.

## 2. Materials and Methods

This study was designed as a single‐center, retrospective, and cross‐sectional study. Patients aged 18 with CS or SCS to 70 years who were diagnosed were included. The diagnosis of CS was established based on clinical features, physical examination findings, and biochemical evaluation results, in accordance with the latest Endocrine Society clinical practice guidelines [2].

As first‐line tests, midnight salivary cortisol (at least two measurements), 24‐h UFC (at least two measurements), and the 1‐mg DST were performed for all patients. CS diagnosis was confirmed in individuals with at least two different positive first‐line test results, followed by additional testing to determine the underlying cause of CS. The cut‐off values for plasma cortisol after 1 mg DST were 1.8 mcg/dL, for midnight salivary cortisol 0.27 mcg/dL, and for 24‐h UFC 130 mcg/day.

For etiological evaluation, morning plasma ACTH levels were measured on at least two separate days in patients diagnosed with CS. Patients with ACTH levels < 5 ng/L were classified as ACTH‐independent CS. Patients with ACTH levels > 20 ng/L were classified as ACTH‐dependent CS. For patients with ACTH levels between 5 and 20 ng/L, additional tests were conducted to determine the etiology: (1) Re‐evaluation of ACTH levels after 4–6 weeks. (2) DHEAS measurement (low DHEAS was consistent with ACTH‐independent CS, whereas normal or increased DHEAS was suggestive of ACTH‐dependent CS). (3) For cases where ACTH‐dependent and independent CS differentiation remained unclear after three repeated ACTH measurements, a CRH or desmopressin stimulation test was performed. Non‐responsive patients were classified as ACTH‐independent and responsive patients were classified as ACTH‐dependent CS. Diagnoses were supported by imaging methods, and when necessary, inferior petrosal sinus sampling (IPSS). Diagnoses were confirmed by postoperative pathology results.

In the early postoperative period, remission was defined as serum cortisol levels below 1.8 mcg/dL, serum cortisol levels lower than 5 mcg/dL at the third postoperative month, at least two normal UFC measurements, 1 mg DST result below 1.8 mcg/dL, and clinically compatible remission status.

The diagnosis of SCS was based on the presence of an incidentally detected adrenal lesion with imaging characteristics suggestive of an adenoma, biochemical confirmation of ACTH‐independent autonomous cortisol hypersecretion, and the absence of a typical CS clinical presentation.

Exclusion criteria included patients with active gastrointestinal bleeding, chronic kidney disease, chronic liver disease, hyperthyroidism, morbid obesity, chronic alcohol consumption, use of medications significantly affecting liver and renal function, or any form of exogenous steroid use.

A total of 229 individuals participated in the study, including 151 patients and 78 healthy hospital employees. Between January 1, 2017, and September 1, 2022, 86 patients diagnosed with CS (52 ACTH‐dependent, 34 ACTH‐independent) and 65 patients diagnosed with SCS were enrolled from the Endocrinology and Metabolic Diseases Clinic of a tertiary training and research hospital in Istanbul, Turkey. Of the participants, 185 (80.8%) were female and 44 (19.2%) were male. The control group consisted of hospital employees who were volunteers with no known medical conditions. Clinical and laboratory data were retrieved from electronic medical records.

Patients were categorized into three groups: the CS group (patients diagnosed with CS), the SCS group (patients diagnosed with SCS), and the healthy control group. The CS group was further categorized into ACTH‐dependent and ACTH‐independent CS. All ACTH‐dependent CS cases resulted from pituitary adenomas, and all ACTH‐independent CS cases resulted from adrenal adenomas.

Demographic data and comorbidities potentially associated with CS (such as diabetes mellitus, hypertension [HT], hyperlipidemia [HL], obesity, ischemic cardiovascular disease [ICVD], and osteoporosis [OP]) were recorded.

To evaluate differences in the BUN/creatinine ratio across study groups and postoperative changes in CS patients, plasma BUN and creatinine levels were recorded in mg/dL for all patients, and the BUN/creatinine ratio was calculated. The calculated BUN/creatinine values were compared between the patient groups and the control group, as well as among the patient groups. In CS patients, preoperative measurements were obtained during the diagnostic work‐up prior to surgery, and postoperative measurements were obtained within the first 72 h after surgery. All laboratory tests in this study were conducted in a single laboratory using standardized equipment and reagents. Blood samples were collected in separator gel tubes with yellow caps, allowed to stand at room temperature for 30 min, and then centrifuged at 1000 g for 20 min. Urea was analyzed using the urease method, and creatinine was measured using the Jaffe alkaline picrate method on the Cobas 8000 c702 analyzer (Roche Diagnostics, Mannheim, Germany) from the obtained serum. Plasma ACTH, serum cortisol, and salivary cortisol were analyzed using the electrochemiluminescence immunoassay method on the Cobas 8000 e 602 module (Roche Diagnostics, Mannheim, Germany). UFC levels were measured using the Maglumi 2000 immunoassay analyzer (Snibe, Shenzhen, China). Additionally, in CS patients, the correlation between the diameter of the adenoma causing hypercortisolism (pituitary or adrenal) and the BUN/creatinine ratio was evaluated. The largest measured diameter of pituitary and adrenal adenomas was recorded in millimeters (mm).

## 3. Statistical Analysis

The sample size was calculated as 64 using the G × Power 3.1.9.7 program (Franz Faul, Germany). Statistical analyses were conducted using SPSS version 17.0. The normality of variable distributions was assessed using histogram plots and the Kolmogorov–Smirnov test. Descriptive statistics were presented as mean, standard deviation, median, minimum, and maximum values.

Categorical variables were compared using the Pearson chi‐square test. Preoperative and postoperative BUN/creatinine ratio values in CS patients were compared using paired analyses. In cases where the data did not follow a normal distribution, the Mann–Whitney *U* test was applied for two‐group comparisons, while the Kruskal–Wallis test was used for comparisons involving more than two groups. Spearman correlation analysis was performed to examine the relationships between measured variables across groups.

Multivariable linear regression analyses were performed to evaluate the independent association between hypercortisolism status (CS, SCS, or combined) and the preoperative BUN/creatinine ratio. Primary regression models were adjusted for age and sex to account for potential demographic confounding. HT, diabetes mellitus, and other metabolic comorbidities were not included in the primary multivariable models because the control group consisted of metabolically healthy individuals by study design, resulting in limited or no variability for these variables across comparison groups. Therefore, primary adjusted analyses focused on demographic covariates.

Threshold values for blood parameters and various test parameters in predicting patient and control groups were analyzed using receiver operating characteristic (ROC) analysis. Analyses were performed using available complete observations for the variables of interest; missing values were not imputed. A *p*‐value < 0.05 was considered statistically significant.

## 4. Findings

Baseline demographic characteristics and comorbidity profiles of the study groups are presented in Table 1. When comparing gender distribution and age across groups, a statistically significant difference was found for age, with the SCS group having a significantly higher mean age than the other groups (*p* < 0.001).

**TABLE 1:** Comparison of demographic characteristics and comorbidity distributions across groups (mean [standard deviation] or number [percentage]).

	**Diagnosis**						**p** **(overall)**	**p** ^**1**^	**p** **2**	**p** ^**3**^	**p** ^**4**^
		**CS (overall)**	**ACTH-dependent CS**	**ACTH-independent CS**	**SCS**	**Controls**					
		***n* (%)**	***n* (%)**	***n* (%)**	***n* (%)**	***n* (%)**					

Sex	Male	16 (18.6)	10 (19.2)	6 (17.6)	9 (13.8)	19 (24.4)	0.459^a^				
	Female	70 (81.4)	42 (80.8)	28 (82.4)	56 (86.2)	59 (75.6)					
Age (years)		50.91 ± 10.57	49.88 ± 11.99	52.47 ± 7.83	60.23 ± 8.92	45.1 ± 12.48	< 0.001**^b^**				
	Diabetes mellitus	34 (39.5)	17 (32.7)	17 (50)	27 (41.5)	—		0.804	0.110	0.330	0.360
	Hypertension	48 (55.8)	21 (40.4)	27 (79.4)	45 (69.2)	—		0.094	**< 0.001**	**0.002**	0.214
	Hyperlipidemia	22 (25.6)	13 (25)	9 (26.5)	18 (27.7)	—		0.772	0.876	0.743	0.885
	Obesity	11 (12.8)	4 (7.7)	7 (20.6)	8 (12.3)	—		0.927	0.081	0.417	0.225
	Ischemic heart disease	8 (9.3)	1 (1.9)	7 (20.6)	6 (9.2)	—		0.983	**0.003**	0.098	0.081
	Osteoporosis	18 (20.9)	7 (13.5)	11 (32.4)	14 (21.5)	—		0.929	**0.036**	0.264	0.185

*Note:* not assessed in controls. *p* (overall), overall between‐group comparison for demographic variables; *p* ^1^–*p* ^4^, post hoc *p* values for pairwise comparisons of comorbidities; *p* ^1^, CS vs. SCS; *p* ^2^, ACTH‐dependent CS vs. ACTH‐independent CS; *p* ^3^, ACTH‐dependent CS vs. SCS; *p* ^4^, ACTH‐independent CS vs. SCS; CS, Cushing’s syndrome; SCS, subclinical Cushing’s syndrome; ACTH, adrenocorticotropic hormone. Bold values indicate statistical significance (*p* < 0.05).

^a^Chi‐square test.

^b^Kruskal–Wallis test.

The prevalence of HT, ICVD, and OP was significantly higher in ACTH‐independent CS patients compared to ACTH‐dependent CS patients (*p*‐values: < 0.001, 0.003, and 0.036, respectively). Additionally, the prevalence of HT was significantly higher in SCS patients compared to ACTH‐dependent CS patients (*p* = 0.002).

The BUN/creatinine ratio was found to be higher in women than in men within the SCS and control groups (*p*‐values: 0.020 and 0.024, respectively).

A comparison of BUN/creatinine ratios between patient groups and the control group is presented in Table 2. The BUN/creatinine ratio was significantly higher in CS, ACTH‐independent CS, and SCS groups compared to the control group (*p*‐values: 0.026, 0.027, and < 0.001, respectively). These findings were further evaluated using adjusted multivariable analyses. However, the mean BUN/creatinine ratio in the ACTH‐dependent CS group was higher than in the control group, though this difference did not reach statistical significance (*p* = 0.121). Additionally, in unadjusted analyses, the SCS group exhibited a significantly higher BUN/creatinine ratio compared to the CS group (*p* = 0.024).

**TABLE 2:** Comparison of BUN/creatinine ratios among groups.

	_**Total CS Group**_		_**ACTH-dependent CS**_		_**ACTH-independent CS**_		_**SCS**_		_**Control**_		**p**	**p** ^**1**^	**p** ^**2**^	**p** ^**3**^	**p** ^**4**^	**p** ^**5**^
	_**Mean ± SD**_	_**Median (Min-Max)**_	_**Mean ± SD**_	_**Median (Min-Max)**_	_**Mean ± SD**_	_**Median (Min-Max)**_	_**Mean ± SD**_	_**Median (Min-Max)**_	_**Mean ± SD**_	_**Median (Min-Max)**_						

_BUN/creatinine ratio_	_19.77 ±5.81_	_18,43__(15.83-22.66)_	_19.34 ±5.61_	_17.77__(15.8-22.45)_	_**20.42 ±6.13**_	_19,49__(15.91-22.73)_	_21.57 ±6.11_	_21.51__(17.76-25.11)_	_**17.55 ±5.24**_	_17.18__(13.53-20.95)_	_**< 0.001**_	_**0.026**_	_**0.024**_	_0.121_	_**0.027**_	_**< 0.001**_

*Note:* *p*, Kruskal–Wallis test; *p* ^1^, comparison between total CS group and control (Mann–Whitney *U* test); *p* ^2^, comparison between total CS group and SCS (Mann–Whitney *U* test); *p* ^3^, comparison between ACTH‐dependent CS and control (Mann–Whitney *U* test); *p* ^4^, comparison between ACTH‐independent CS and control (Mann–Whitney *U* test); *p* ^5^, comparison between SCS and control (Mann–Whitney *U* test); ACTH, adrenocorticotropic hormone; min, minimum; max, maximum. Bold values indicate statistical significance (*p* < 0.05).

Abbreviations: BUN, blood urea nitrogen; CS, Cushing’s syndrome; SD, standard deviation; SCS, subclinical Cushing’s syndrome.

The preoperative and postoperative BUN/creatinine ratio values for remission evaluation in CS patients are shown in Table 3. Postoperative values for CS and its ACTH‐dependent CS subgroup were significantly lower than preoperative values (*p* < 0.001). However, in the ACTH‐independent CS subgroup, although the ratio decreased postoperatively, the change was not statistically significant (*p* = 0.107).

**TABLE 3:** Comparison of preoperative and postoperative BUN/creatinine ratios.

	**Preoperative BUN/creatinine**		**Postoperative BUN/creatinine**		**Percentage change (%)**	**p**
	**Mean ± SD**	**Median (min–max)**	**Mean ± SD**	**Median (min–max)**	**(%)**	

Total CS group	19.77 ± 5.81	18.43 (15.83–22.66)	16.17 ± 5.83	14.76 (12–20.91)	18.2	**< 0.001**
ACTH‐dependent CS	19.34 ± 5.61	17.77 (15.8–22.45)	14.63 ± 5.29	13.85 (11.68–15.93)	24.35	**< 0.001**
ACTH‐independent CS	20.42 ± 6.13	19.49 (15.91–22.73)	18.65 ± 5.89	18.14 (14.26–23.68)	8.67	0.107

*Note:* ACTH, adrenocorticotropic hormone; min, minimum; max, maximum. Bold values indicate statistical significance (*p* < 0.05).

Abbreviations: BUN, blood urea nitrogen; CS, Cushing’s syndrome; SCS, subclinical Cushing’s syndrome; SD, standard deviation.

To determine whether the observed differences in preoperative BUN/creatinine ratio were independent of demographic differences between groups, multivariable linear regression analyses adjusted for age and sex were performed (Table 4). After adjustment, preoperative BUN/creatinine ratio remained significantly higher in patients with CS, SCS, and in the combined CS + SCS group compared with controls (*p* = 0.015, *p* = 0.009, and *p* = 0.002, respectively). Additional multivariable regression analyses comparing CS and SCS are presented in Supporting Table S2.

**TABLE 4:** Multivariable linear regression analysis of preoperative BUN/creatinine ratio.

	**CS vs. control**		**SCS vs. control**		**CS + SCS vs. control**	
	**Beta (SE)**	**p**	**Beta (SE)**	**p**	**Beta (SE)**	**p**

CS	2.135 (0.866)	**0.015**				
SCS			2.704 (1.018)	**0.009**		
CS + SCS					2.493 (0.796)	**0.002**
Age (years)	0.023 (0.037)	0.538	0.090 (0.041)	**0.032**	0.077 (0.031)	**0.014**
Male sex	1.884 (1.055)	**0.076**	3.726 (1.156)	**0.002**	2.572 (0.945)	**0.007**

*Note:* Beta, unstandardized regression coefficient. Bold values indicate statistical significance (*p* < 0.05).

Abbreviations: BUN, blood urea nitrogen; CS, Cushing’s syndrome; SCS, subclinical Cushing’s syndrome; SE, standard error.

The relationship between preoperative BUN/creatinine ratio and CS diagnostic tests (including basal plasma cortisol, 1‐mg DST, 2‐mg DST for 2 days, 24‐h UFC, midnight salivary cortisol, and midnight serum cortisol) was analyzed across all patient groups (Table 5). In all CS patients, a significant correlation was found between BUN/creatinine ratio and 1‐mg DST and midnight serum cortisol (*p*‐values: 0.041 and 0.049, respectively). In the ACTH‐dependent CS group, a positive correlation was observed between BUN/creatinine ratio and basal plasma cortisol, 1‐mg DST, and midnight serum cortisol (*p*‐values: 0.047, 0.038, and 0.004, respectively). However, no significant correlation was found between BUN/creatinine ratio and CS diagnostic tests in the ACTH‐independent CS and SCS groups.

**TABLE 5:** Analysis of the relationship between BUN/creatinine ratio and Cushing’s syndrome diagnostic tests.

		**BUN/creatinine ratio**			
		**Total CS group**	**ACTH-dependent CS**	**ACTH-independent CS**	**SCS**

Basal plasma cortisol (mcg/dL)	r	0.190	0.277	0.153	0.063
	*p*	0.079	**0.047**	0.386	0.616

1‐mg DST (mcg/dL)	r	0.222	0.291	0.134	0.079
	*p*	**0.041**	**0.038**	0.450	0.534

2‐Day 2‐mg DST (mcg/dL)	r	0.125	0.032	0.205	−0.091
	*p*	0.265	0.830	0.260	0.479

24‐h urinary free cortisol (mcg/day)	r	0.028	−0.054	0.242	−0.247
	*p*	0.806	0.714	0.175	0.051

Midnight plasma cortisol (mcg/dL)	r	0.223	0.409	0.228	0.176
	*p*	**0.049**	**0.004**	0.210	0.175

Midnight salivary cortisol (mcg/dL)	r	0.220	0.297	0.244	0.016
	*p*	0.064	0.059	0.186	0.899

*Note:* *p*, Spearman correlation test; ACTH, adrenocorticotropic hormone; mcg, microgram; dL, deciliter. Bold values indicate statistical significance (*p* < 0.05).

Abbreviations: BUN, blood urea nitrogen; CS, Cushing’s syndrome; DST, dexamethasone suppression test; SCS, subclinical Cushing’s syndrome.

The relationship between adenoma diameter and BUN/creatinine ratio in ACTH‐dependent and ACTH‐independent CS groups is presented in Table 6. A statistically significant correlation was observed between pituitary adenoma diameter and BUN/creatinine ratio (*p* = 0.036), whereas no significant correlation was found between adrenal adenoma diameter and BUN/creatinine ratio (*p* = 0.922).

**TABLE 6:** Analysis of the relationship between BUN/creatinine ratio and adenoma diameter.

	**Preoperative BUN/creatinine ratio**	

Pituitary adenoma diameter in ACTH‐dependent CS	r	0.314
	*p*	**0.036**

Adrenal adenoma diameter in ACTH‐independent CS	r	0.018
	*p*	0.922

*Note:* Bold values indicate statistical significance (*p* < 0.05). *p*, Spearman correlation test; ACTH, adrenocorticotropic hormone.

Abbreviations: BUN, blood urea nitrogen; CS, Cushing’s syndrome.

The discriminatory performance of BUN/creatinine ratio between CS, ACTH‐dependent CS, ACTH‐independent CS, and SCS groups, along with cut‐off values, was analyzed as shown in Table 7. A BUN/creatinine ratio cut‐off value of > 15.34 was associated with the CS group, with 81.40% sensitivity and 38.46% specificity (*p* = 0.026). For the ACTH‐independent CS group, a cut‐off of > 13.67 yielded 97.06% sensitivity and 26.92% specificity (*p* = 0.027). For the SCS group, a cut‐off of > 19.8 provided 63.08% sensitivity and 70.51% specificity (*p* < 0.001). No significant cut‐off value was identified for the ACTH‐dependent CS group.

**TABLE 7:** Diagnostic cutoff values for BUN/creatinine ratio based on ROC analysis.

BUN/Creatinine ratio	Cutoff	AUC	Std. Error	*p*	95% confidence interval		Sensitivity (%)	Specificity (%)	PPV (%)	NPV (%)
					Lower limit	Upper limit				
Total CS group	> 15.34	0.601	0.044	**0.026**	0.514	0.687	81.40	38.46	61.54	65.22
ACTH‐dependent CS		0.580	0.051	0.121	0.481	0.680				
ACTH‐independent CS	> 13.67	0.631	0.055	**0.027**	0.525	0.738	97.06	26.92	36.67	95.45
SCS	> 19.8	0.695	0.044	**<** **0.001**	0.609	0.781	63.08	70.51	64.06	69.62

*Note:* ACTH, adrenocorticotropic hormone; Std, standard. Bold values indicate statistical significance (*p* < 0.05).

Abbreviations: AUC, area under the curve; BUN, blood urea nitrogen; CS, Cushing’s syndrome; NPV, negative predictive value; PPV, positive predictive value; SCS, subclinical Cushing’s syndrome.

## 5. Discussion

Due to the wide range of clinical manifestations in CS, distinguishing true cases that require further evaluation from the large number of suspected cases is of great importance [9]. Therefore, screening tests should not be expensive, complex, or difficult to perform. In addition, they should have acceptable sensitivity and specificity. An ideal test should be easily accessible, simple to perform, and cost‐effective [10]. BUN and serum creatinine levels are low‐cost, routinely used biochemical markers that can be performed even in primary healthcare settings.

Previous studies have shown that the BUN/creatinine ratio increases in conditions involving catabolism and exogenous steroid use [11]. More recently, studies have supported the use of the BUN/creatinine ratio as a biomarker of catabolism, particularly in correlation with muscle breakdown [8, 12].

In our study, the BUN/creatinine ratio at the time of diagnosis was found to be significantly higher in patients with CS and SCS compared to the control group, allowing for the exploration of cut‐off values. This difference was more pronounced in SCS patients. Importantly, this association persisted after adjustment for age and sex in multivariable analyses, indicating that elevated BUN/creatinine ratios are independently associated with hypercortisolism rather than being solely explained by demographic differences. Exploratory subgroup analyses also showed a significant age‐ and sex‐adjusted association between preoperative BUN/creatinine ratio and ACTH‐independent CS, whereas no significant association was observed for ACTH‐dependent CS (Supporting Table S1). Additionally, a cut‐off value was identified for the ACTH‐independent CS subgroup. Overall, the adjusted analyses support a consistent association between hypercortisolism and higher BUN/creatinine ratio across the studied subgroups.

In our study, the BUN/creatinine ratio for the healthy population was calculated as 17.55 ± 5.24. At the time of diagnosis, the BUN/creatinine ratio was 19.77 ± 5.81 in the CS group, 19.34 ± 5.61 in the ACTH‐dependent CS group, 20.42 ± 6.13 in the ACTH‐independent CS group, and 21.57 ± 6.11 in the SCS group. Comparisons with the control group revealed statistically significant differences, with values being higher in the CS, ACTH‐independent CS, and SCS groups. To the best of our knowledge, no previous study has investigated the BUN/creatinine ratio in CS or SCS, making direct comparisons impossible. This highlights the novelty of our research, which stands as the first study to explore this association.

A study conducted in Japan evaluating the role of the cortisol/ACTH ratio in CS diagnosis compared cortisol/ACTH ratios with 1‐mg DST results. In cases of ACTH‐independent CS, a positive correlation was found between the cortisol/ACTH ratio and 1‐mg DST results [13]. Similarly, when examining the BUN/creatinine ratio used in our study, a positive correlation was observed between the BUN/creatinine ratio and 1‐mg DST (*r* = 0.222, *p* = 0.041) as well as midnight serum cortisol (*r* = 0.223, *p* = 0.049) in the CS group. In the ACTH‐dependent CS group, the BUN/creatinine ratio showed a positive correlation with basal plasma cortisol (*r* = 0.277, *p* = 0.047), 1‐mg DST (*r* = 0.291, *p* = 0.038), and midnight serum cortisol (*r* = 0.409, *p* = 0.004). These correlations, along with the identified cut‐off values for different groups, suggest that the BUN/creatinine ratio may be considered an adjunctive parameter in the evaluation of patients with CS.

The change in preoperative and postoperative BUN/creatinine ratios was found to be statistically significant in the CS and ACTH‐dependent CS groups. In the ACTH‐independent CS group, no statistically significant change was observed; however, considering the significance in other groups, it is anticipated that a larger sample size might reveal a significant decrease in BUN/creatinine ratio with treatment in this group as well. These findings indicate that the BUN/creatinine ratio changes after treatment in CS, particularly in ACTH‐dependent CS, although these findings should be considered exploratory.

A study published in 2022 evaluated the relationship between adenoma size and biochemical test results in ACTH‐dependent CS. In this retrospective study, which included 105 patients, participants were classified into a microadenoma group (80 cases; mean diameter: 5.2 ± 2.2 mm) and a macroadenoma group (25 cases; mean diameter: 18.0 ± 7.7 mm). Although 1‐mg DST and UFC values were higher in the macroadenoma group, the differences were not statistically significant. However, ACTH levels were significantly higher in the macroadenoma group. No correlation was found between adenoma size and cortisol secretion levels or the clinical severity of hypercortisolism [14]. In contrast, our study demonstrated a statistically significant and positive correlation between adenoma size and the BUN/creatinine ratio in patients with ACTH‐dependent CS.

In the literature, commonly used diagnostic tests have established cut‐off values as follows: For 1‐mg DST, a cut‐off of 1.8 mcg/dL yields a sensitivity of 95% and a specificity of 80%. For 2‐‐mg DST, using the same cutoff value of 1.8 mcg/dL results in a sensitivity of 95% and a specificity of 70%. Midnight serum cortisol, when measured during sleep with a cut‐off of 1.8 mcg/dL, has been reported to have a sensitivity of 100%. However, in larger and more recent studies, specificity was found to be 20%. For midnight salivary cortisol, a cut‐off of 0.145 mcg/dL (4 nmol/L) has been determined, with studies from different countries reporting a sensitivity of 92%–100% and a specificity of 93%–100%. The CRH‐dexamethasone test, with a cutoff of 1.8 mcg/dL, has been reported to have a sensitivity of 98% and a specificity of 60% [3].

**FIGURE 1:**
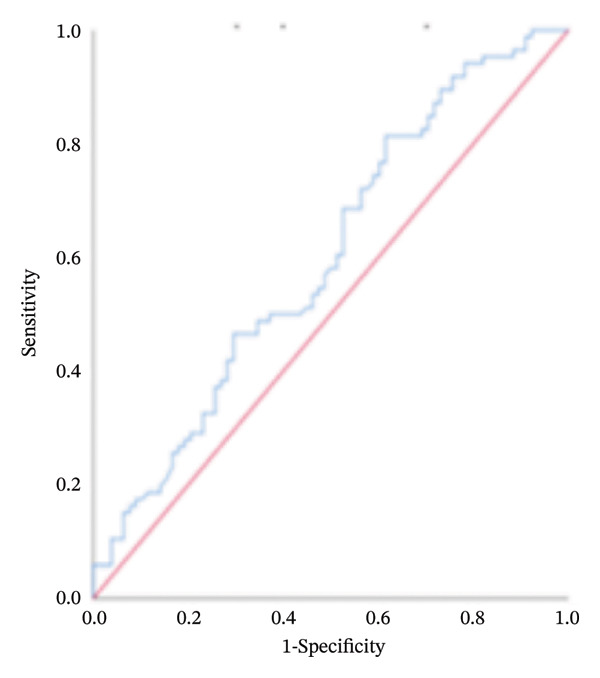
ROC analysis for preoperative BUN/creatinine ratio in Cushing’s syndrome. Abbreviations: ROC, receiver operating characteristic; BUN, blood urea nitrogen.

**FIGURE 2:**
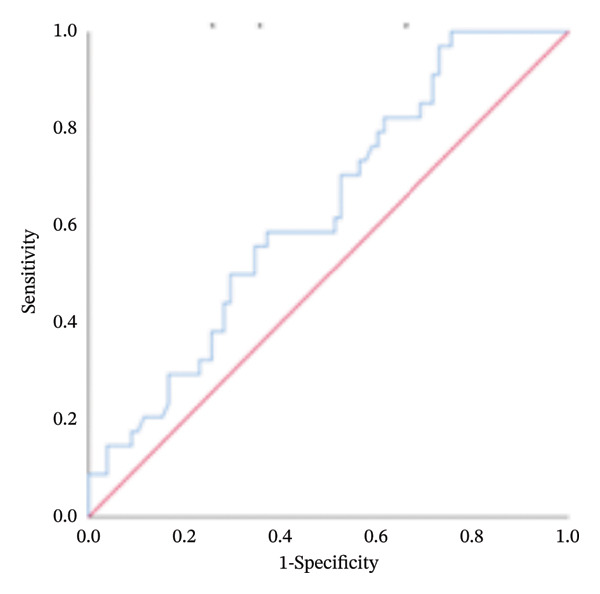
ROC analysis for preoperative BUN/creatinine ratio in ACTH‐independent Cushing’s syndrome. Abbreviations: ROC, receiver operating characteristic; BUN, blood urea nitrogen; ACTH, adrenocorticotropic hormone.

**FIGURE 3:**
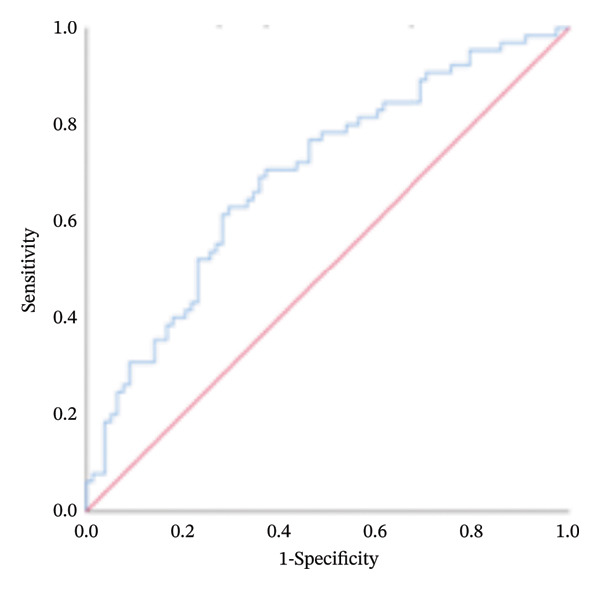
ROC analysis for BUN/creatinine ratio in subclinical Cushing’s syndrome. Abbreviations: ROC, receiver operating characteristic; BUN, blood urea nitrogen.

In our study, the cut‐off values for preoperative BUN/creatinine ratio were explored using ROC analysis in CS and SCS as follows: > 15.34 for CS (*p* = 0.026, sensitivity: 81.40%, specificity: 38.46%), > 13.67 for ACTH‐independent CS (*p* = 0.027, sensitivity: 97.06%, specificity: 26.92%), and > 19.8 for SCS (*p* < 0.001, sensitivity: 63.08%, specificity: 70.51%). Since no statistically significant difference was detected in the preoperative BUN/creatinine ratio for the ACTH‐dependent CS group, ROC analysis was not performed for this subgroup. As there are no similar studies in the literature, the obtained cutoff values could not be compared. Our findings suggest that the BUN/creatinine ratio may show relatively high sensitivity for CS and the ACTH‐independent CS subgroup; however, given the limited specificity observed, its discriminatory performance and potential clinical utility should be interpreted cautiously and considered exploratory (see Figures 1, 2, and 3).

One limitation of our study is that the sample consisted exclusively of pituitary adenoma‐derived cases in the ACTH‐dependent CS group and adrenal adenoma‐derived cases in the ACTH‐independent CS group. In addition, postoperative changes in the BUN/creatinine ratio may reflect perioperative fluid shifts, nutritional factors, or metabolic stress rather than cortisol normalization alone and should therefore be interpreted cautiously. Finally, in the control group, the absence of comorbidities and regular medication use was confirmed based on medical history and electronic medical records; however, no additional screening tests were performed. BMI, medication categories, and additional biochemical parameters were not consistently available in the retrospective records and therefore could not be included in the baseline characteristics table. Further studies with larger sample sizes, encompassing all etiologies, are needed to provide a more comprehensive evaluation.

## 6. Conclusion

Our study highlights the potential of the BUN/creatinine ratio as an adjunctive parameter associated with CS, its ACTH‐independent CS subgroup, and SCS. At the time of diagnosis, the BUN/creatinine ratio differed between these groups, while postoperative comparisons demonstrated a statistically significant decrease in CS and ACTH‐dependent CS. However, these postoperative findings should be interpreted cautiously and considered exploratory. Importantly, this association persisted after adjustment for age and sex, supporting an independent relationship between hypercortisolism and the BUN/creatinine ratio.

Moreover, the observed correlation between the BUN/creatinine ratio and pituitary adenoma size in ACTH‐dependent CS indicates a potential association between this ratio and adenoma size.

These findings underscore the potential role of the BUN/creatinine ratio as a complementary parameter reflecting CS status and postoperative changes, particularly in cases where conventional tests cannot be fully implemented or interpreted.

To clarify the clinical relevance of these findings, large‐scale, multicenter, prospective studies are essential, aiming to establish cut‐off values with improved sensitivity and specificity. Additionally, further research is needed to explore the association between the BUN/creatinine ratio and adenoma size, including differences between microadenomas and macroadenomas, as well as its behavior in special patient populations (see Figures 1, 2 and 3).

## Author Contributions

C. Yarkutay Turkkan: conceptualization, data curation, investigation, and writing–original draft.

M.M. Canat: formal analysis, data curation, and writing–review and editing.

H. Erhan Gol: data curation and investigation.

Y. Altuntas: supervision and writing–review and editing.

## Funding

This research did not receive any specific grant from funding agencies in the public, commercial, or not‐for‐profit sectors.

## Disclosure

All authors confirm that they meet the criteria for authorship, have contributed significantly to the work, have approved the final version of the manuscript, and agree with its submission to International Journal of Endocrinology.

## Ethics Statement

The study was approved by the Ethics Committee of Şişli Hamidiye Etfal Training and Research Hospital (Approval No: 2200, Date: 13.12.2022) and conducted in accordance with the principles of the Declaration of Helsinki.

## Conflicts of Interest

The authors declare no conflicts of interest.

## Supporting Information

Additional supporting information can be found online in the Supporting Information section.

## Supporting information


**Supporting Information** Supporting Table S1: Multivariable linear regression analysis of the preoperative BUN/creatinine ratio in ACTH subgroups. Supporting Table S2: Multivariable linear regression analysis of the preoperative BUN/creatinine ratio: CS vs. SCS.

## Data Availability

The data that support the findings of this study are available from the corresponding author upon reasonable request and with permission from the affiliated institution.
