# Supplementary material for: Blood Urea Nitrogen/Creatinine Ratio in Cushing’s Syndrome: Associations With Disease Status and Postoperative Changes
Source: Int J Endocrinol. 2026 Jun 12;2026:2517065. doi: 10.1155/ije/2517065 (PMC13263411; doi:10.1155/ije/2517065)
Supplement: Supplementary file 1 — Supporting Information Supporting Table S1: Multivariable linear regression analysis of the preoperative BUN/creatinine ratio in ACTH subgroups. Supporting Table S2: Multivariable linear regression analysis of the preoperative BUN/creatinine ratio: CS vs. SCS. [file IJE-2026-2517065-s001.docx]

**Supplementary Table S1:**Multivariable Linear Regression Analysis of Preoperative BUN/Creatinine Ratio in ACTH Subgroups

|  | ACTH-dependent CS vs Control | | ACTH-independent CS vs Control | |
| --- | --- | --- | --- | --- |
|  | Beta (SE) | p | Beta (SE) | p |
| ACTH-dependent CS | 1.480 (1.015) | 0.147 |  |  |
| ACTH-independent CS |  |  | 2.659 (1.209) | **0.030** |
| Age (years) | -0.008 (0.044) | 0.846 | 0.028 (0.047) | 0.555 |
| Male sex | 1.513 (1.128) | 0.182 | 3.235 (1.240) | **0.010** |

Abbreviations: CS, Cushing’s syndrome; BUN, blood urea nitrogen; ACTH, adrenocorticotropic hormone; Beta, unstandardized regression coefficient; SE, standard error..

**Supplementary Table S2:** Multivariable Linear Regression Analysis of Preoperative BUN/Creatinine Ratio: CS vs SCS

|  | **Beta (SE)** | **p** |
| --- | --- | --- |
| CS | -0.814 (1.092) | 0.457 |
| Age | 0.059 (0.053) | 0.267 |
| Male sex | 2.205 (1.279) | 0.087 |
| Obesity | 0.731 (1.488) | 0.624 |
| Diabetes mellitus | 2.028 (1.087) | 0.064 |
| Hypertension | 1.093 (1.217) | 0.370 |

Abbreviations: CS, Cushing’s syndrome; SCS, subclinical Cushing’s syndrome; BUN, blood urea nitrogen; Beta, unstandardized regression coefficient; SE, standard error.

Note: Model adjusted for age, sex, obesity, diabetes mellitus, and hypertension.
